# Worse becomes the worst: obesity inequality, its determinants and policy options in Iran

**DOI:** 10.3389/fpubh.2024.1225260

**Published:** 2024-02-07

**Authors:** Fatemeh Toorang, Parisa Amiri, Abolghassem Djazayery, Hamed Pouraram, Amirhossein Takian

**Affiliations:** ^1^Department of Community Nutrition, School of Nutritional Sciences and Dietetics, Tehran University of Medical Sciences, Tehran, Iran; ^2^Cancer Research Center, Cancer Institute of Iran, Tehran University of Medical Science, Tehran, Iran; ^3^Department of Medical and Surgical Sciences, University of Bologna, Bologna, Italy; ^4^Research Center for Social Determinants of Health, Research Institute for Endocrine Sciences, Shahid Beheshti University of Medical Sciences, Tehran, Iran; ^5^Departments of Global Health and Public Policy, School of Public Health, Tehran University of Medical Sciences, Tehran, Iran; ^6^Department Health Management, Policy, and Economics, School of Public Health, Tehran University of Medical Sciences, Tehran, Iran; ^7^Health Equity Research Center (HERC), Tehran University of Medical Sciences, Tehran, Iran

**Keywords:** health policy, health inequality, obesity, overweight, qualitative study

## Abstract

**Background:**

This tracked obesity inequality and identified its determinants among the population of Iran. In addition, it examined the impact of implemented policies on these inequalities.

**Methods:**

This study was performed in two phases. First, we conducted a rapid review of the disparity in obesity prevalence in Iran. Then we investigated the main determinants of this inequality in a qualitative study. In addition, we examined Iran’s policies to deal with obesity from the perspective of equality. We conducted 30 Semi-structured interviews with various obesity stakeholders selected through a purposive snowball sampling method between November 25, 2019, and August 5, 2020. In the inductive approach, we used the content analysis method based on the Corbin and Status framework to analyze the data using MAXQDA-2020. The consolidating criteria for reporting a Qualitative Study (COREQ-32) were applied to conduct and report the study.

**Results:**

Inequalities in the prevalence of obesity in terms of place of residence, gender, education, and other socioeconomic characteristics were identified in Iran. Participants believed that obesity and inequality are linked through immediate and intermediate causes. Inequality in access to healthy foods, physical activity facilities, and health care are the immediate causes of this inequality. Intermediate factors include inequality against women, children, and refugees, and inequality in access to information, education, and financial resources. Policymakers should implement equity-oriented obesity control policies such as taxing unhealthy foods, subsidizing healthy foods, providing healthy and free meals in schools, especially in disadvantaged areas, and providing nutrient-rich foods to low-income families. Also, environmental re-engineering to increase opportunities for physical activity should be considered. Of course, for the fundamental reduction of these inequalities, the comprehensive approach of all statesmen is necessary.

**Conclusion:**

Obesity inequality is a health-threatening issue in Iran that can prevent achieving human development goals. Targeting the underlying causes of obesity, including inequalities, must be considered.

## Highlights

- Inequality in obesity prevalence is reported in several high and low-income countries, including Iran. Obesity prevalence is disparate based on accommodation, gender, education, and other socioeconomic characteristics in Iran.
- Obesity and inequality are linked through immediate and intermediate causes. Inequality in access to healthy foods, physical activity facilities, and health care is the immediate cause of this inequality. Of course, these immediate factors result from intermediate factors, including inequality against women, children, and refugees, and inequality in access to information and education, and financial resources.
- Obesity control policies should pay more attention to the fundamental promotors of obesity inequality. Policymakers should implement equity-oriented obesity control policies such as taxing unhealthy foods, subsidizing healthy foods, providing healthy and free meals in schools, especially in disadvantaged areas, and providing nutrient-rich foods to low-income families. Also, environmental re-engineering to increase opportunities for physical activity, especially in marginalized and disadvantaged areas of the country, should be considered

## Introduction

Overweight and obesity are a sweeping public health concern worldwide, and their prevalence has tripled between 1975 and 2016 in most countries, particularly in low- and middle-income countries. In 2016, approximately 2.5 billion adults were overweight or obese. The prevalence of overweight and obesity in men was 39 and 11%, respectively. These figures were 40 and 15% for women that year (1). About 38.9 million children under 5 were overweight or obese in 2020 (2). Obesity, once a problem in high-income countries, has recently increased in prevalence in low- and middle-income countries (3). Most people live in areas where overweight and obese threaten their lives more seriously than underweight (3).

The prevalence of obesity in the Eastern Mediterranean Region (EMRO) is critically high, with more than 45% of men and 50% of women overweight. In addition, obesity is associated with the Human Development Index (HDI) in this region (4). Iran is a country in EMRO that has started an unceasing effort to tackle non-communicable diseases (NCDs) and achieve the Sustainable Development Goals (SDGs) (5). Despite efforts to improve the nutritional status in this country, it seems that there is a significant inequality in the nutritional status of Iranian adults and children.

Inequity is defined as a systematic disparity in health that can be prevented through appropriate policies. Therefore, these differences are considered unfair and unjust (6). Disparities in obesity by gender, socio-economic, and ethnic groups have been reported worldwide and are widening in many populations. Alleviating disparities in obesity is essential to public health because it can describe part of the social gradient in morbidity and mortality (7, 8). And it is an important driver of increasing inequality in health and longevity in the future (7, 8).

The higher prevalence of obesity in lower socio-economic groups puts them in a vicious cycle in which people with lower socioeconomic levels are at greater risk for obesity, and obesity reduces their chances of moving up to a higher social class. It is generally accepted that obese people experience more social exclusion, discrimination, and unemployment. In addition, they earn less and take more time off from work (6). Therefore, obesity prevention policies should ensure that vulnerable people who belong to vulnerable social groups benefit more from our intervention packages (9).

Despite long-term efforts to tackle obesity in Iran, its prevalence has increased in recent years. In addition, there are speculations about the existence of inequality in the prevalence of obesity in Iran. However, no study has investigated the reasons for this disparity. In this way, adding expert opinions and local wisdom to tackle obesity disparities can help design better programs to control this problem. This knowledge is based on the principles of environmental care, their interrelationship with the residential area, and collective responsibility (10). Therefore, this study was designed to track obesity inequalities in Iran. In addition, we aimed to examine the main causes of potential inequality in obesity among the Iranian population and to examine the consequences of policies implemented to fight obesity on this inequality. We hope that it can help to redesign policies in line with equality-oriented policies.

## Methods

### Setting

This paper reports part of the mixed-method prospective policy analysis for obesity prevention and control in Iran. During our journey to improve obesity control in Iran, we observed inequality in the prevalence of obesity in all age groups of Iranians. Therefore, there was a need to investigate the causes of this inequality and how obesity control policies affect inequalities. This article reports our effort to understand the impact of contextual factors and their interaction with obesity inequality in Iran.

In this article, we first conducted a rapid review of studies related to inequality in the prevalence of obesity in Iran, the results of which are shown in Supplementary Table 1. Then, we performed a qualitative study to investigate the factors that create this existing inequality from the view of experts and stakeholders. In addition, we looked at the effects of Iran’s health-related policies on these inequalities. The qualitative part of the research is based on the framework of Corbin and Strauss (11). Moreover, we applied the consolidating criteria for a reporting Qualitative Study to perform and report the results of this study (12). The COREQ-32 item checklist is presented in Supplementary Table 3.

### Rapid review

As mentioned before, we conducted a rapid review in the first phase of our study using methods offered by BMJ and Coc As previously mentioned, we performed a rapid review in the first phase of our study using the methods provided by BMJ and Cochrane (11, 12). First, we prepared a research strategy consulting with some key stakeholders of obesity control and monitoring in Iran. This strategy involves the search for disparities in the prevalence of obesity between different socioeconomic classes, genders, ethnicities, and places of residence. We searched on Google Scholar and Scopus. In addition, we have reviewed national health reports in recent years. We also sought about the difference in the prevalence of obesity in immigrants compared to Iranians. The search strategy was formulated by the FT after discussion with stakeholders and then amended by all members of the research team. Literatures and national reports were studied and needed data were extracted by the FT. Then, the results were reviewed by all authors. The need for some new searches as well as changes to the draft was clarified by the authors. After the requested changes were made, the final report was prepared.

### Interview guide

The interview guide was developed after our rapid literature review on obesity disparities in Iran. We drafted a guide which was reviewed and edited by 5 faculty members. In this interview guide, after introducing the goals of the project, the participants were asked to describe the bellowing questions:

1. Are there any disparities in obesity prevalence in Iran.? If yes, the disparities exist against which groups? (It was a warming question)
2. What are the causes of inequality in the prevalence of obesity in different Iranian groups? (Considering the answer to the first question)
3. What are the effects of obesity control policies or other related policies in Iran on the existing inequality?
4. What are your suggestions for ameliorating the obesity inequalities in Iran?

### Participants and data collection

We conducted 30 semi-structured in-depth interviews between November 25, 2019, and March 18, 2021. The participants were a heterogeneous group consisting of various stakeholders of obesity control in Iran including scientists, government officials, industry, and civil societies. The complete list of participants is presented in Table 1. Purposive sampling was applied to start the interviews with key experts and then continued with the snowball sampling method. Also, some stakeholders were added to the study according to the codes that emerged during the analysis of previous interviews. Interviews were conducted with different stakeholders from different perspectives so that we could understand all the unclear reasons for the inequality in the prevalence of obesity in Iran. Interviews continued until at least one representative from all stakeholder categories was included, and new interviews did not provide additional information on the topic under investigation (13).

**Table 1:** Characteristic of interviewees.

#	Group	Organization	Position	Specialty	Code
1	Academic member	University A in Tehran	Full professor	Epidemiology	AE
2		University A in Tehran	Full professor	Community Nutrition	AN1
3		University B in Tehran	Full professor	Community Nutrition	AN2
4		Research Institute A in Tehran	Researcher	Community Nutrition	AN3
5		University A in Tehran	Full professor	health management	AM
6		University B in Tehran	Assistant professor	food science and technology	AF
7		University A in Tehran	Associate professor	sociology	AS1
8		University C in Tehran	Associate professor	sociology	AS2
9		Research Institute B in Tehran	Assistant professor	physical education	AP1
10		University A in Isfahan	Full professor	pediatrics and clinician	AC
11		university A in Tehran	Associate professor	Health economy	AEC1
12	State organization	Institute of Standards and Industrial Researches	Administrator in a province	Food Technology	ISIR1
13		Ministry of Education	An expert in health administers, the Ministry of education	Education	Edu1
14			An administrator in Mazandaran province	Education	Edu2
15			An administrator in the Kerman province	Education	Edu3
16			A health worker in the Tehran province	Nutrition Science	Edu4
17		Secretariat of the Supreme Council for Health and Food Security	An expert	Physician	SSCHFS
18		Ministry of Health and Medical Education (MoHME)	An administrator	Community Nutrition	MoHME1
19			An administrator	Physician	MoHME2
20			An administrator	Pediatrician	MoHME3
21		a Food and Drug Administration	A clerk	Food technology	FD
22		health administers in the Municipality	A clerk		M1
23		The Islamic Republic of Iran Broadcasting	administrator A	Physician	IRIB1
24			administrator B	Pediatrician	IRIB2
25		Ministry of Interior	An administrator of a health program	Urban planning	MN
26	Industrial company	Food Industry	Manager A	Food industry	FI1
27			Manager B	Management	FI2
28	Non-profit organization	A nonprofit organization on health education	Manager	Nutrition	NPO
29	International organization	International organization A	An expert in the Iran office	Public health in nutrition	INT1
30		International organization B	An expert in the Iran office	NCD control	INT2

The main researcher contacted different stakeholders to explain the purpose and process of the project. If they agreed to participate, an interview was scheduled. The interviews lasted between 30 and 90 min using a semi-structured interview guide including open-ended questions and follow-up questions based on the participants’ answers and positions. Most of the interviews (*n* = 34) were conducted in the participants’ offices in a private setting. Due to the COVID-19 quarantine in the middle of our study, few interviews were conducted by telephone (*n* = 6). During the in-depth interviews, the non-verbal responses and behaviors of the interviewees, as well as their main points of view, were noted and considered in the data analysis. All interviews were recorded and then fully transcribed.

### Data analysis

A deductive content analysis approach was used. Data collection and analysis were done simultaneously according to the qualitative study approaches (14). Before conducting a new interview, the previous interviews were reviewed and the interview guide was modified if necessary so that the new themes could be explored in more depth and detail in the subsequent interviews (13). The analysis team read the transcripts and notes and listened to all audiotapes several times. Further analyses were performed using MAXQDA 2020 for the open, axial, and selective coding phases. The coding process of one-third of the interviews was checked by a qualitative studies expert (PA) to control the coding process. Main themes emerged through the integration of axillary codes. FT and AT discussed the extracted themes and subthemes in several meetings and developed the conceptual framework. Finally, the conceptual framework was reviewed by all authors to ensure the validity of the qualitative analysis and consistency of findings across authors.

## Results

The rapid review shows that there is a significant gender and socio-economic inequality in the nutritional status of Iranian adults and children. Studies have reported differences in the prevalence of obesity in Iran based on place of residence, gender, education, and other socio-economic characteristics (13–17). A systematic review of this issue showed that age, low education level, living in cities, being married, and female gender are associated with the risk of obesity (13). Based on reviewed studies, it could be claimed that the prevalence of obesity in adults of lower socioeconomic status is almost two times in most provinces of Iran (14).

**Figure 1:**
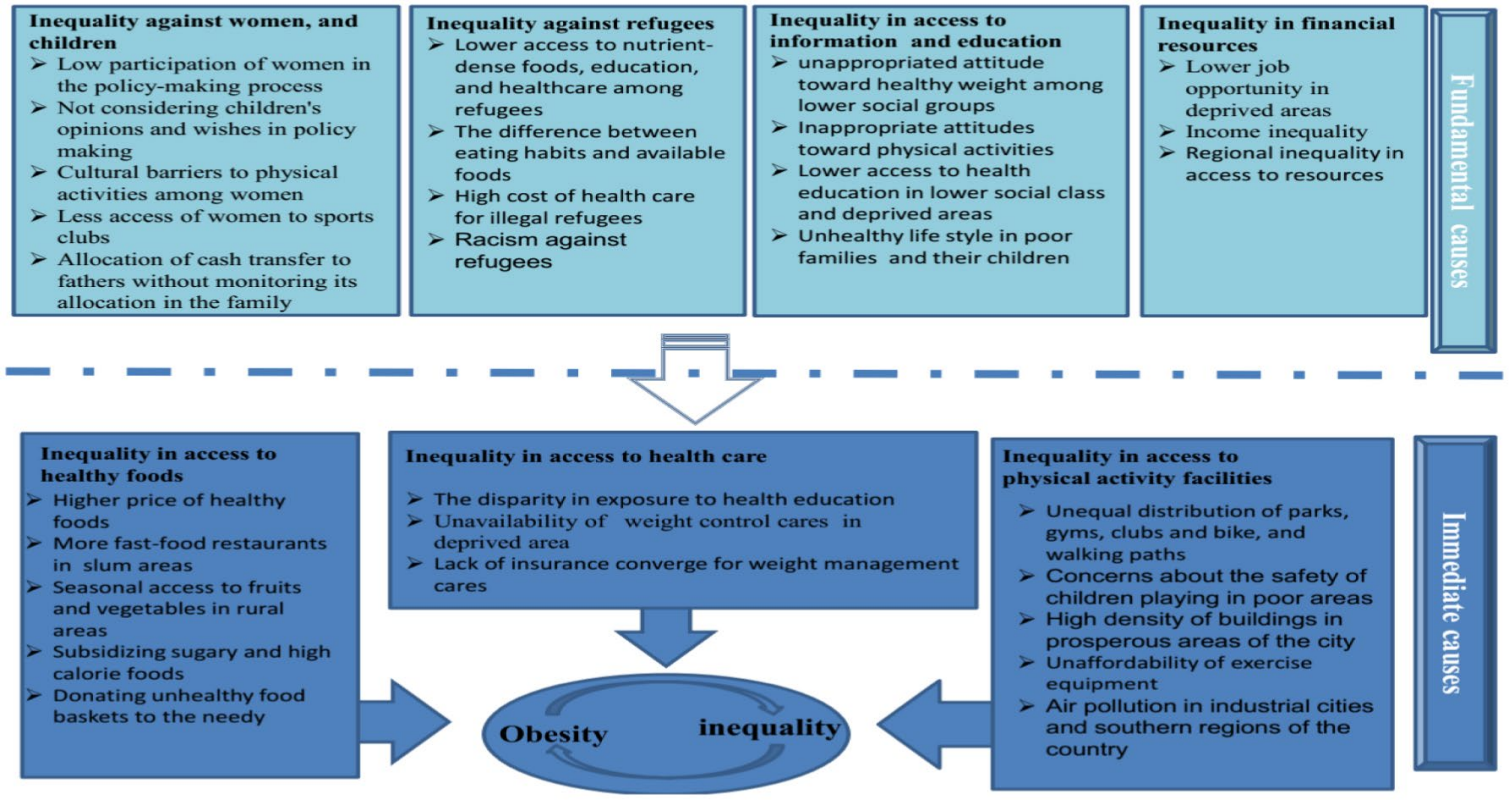
Obesity inequality determinants in Iran.

Based on the participants’ statements, we created a model related to inequality and obesity in Iran, which is shown in Figure 1 and explained in detail below. According to our qualitative content analysis, obesity and inequality are related in Iran. This relationship is defined in two levels of causes, which we divide into immediate and fundamental causes. The interviewees believed that inequality in access to healthy foods, physical activity facilities, and health care are the immediate causes of obesity inequality in Iran. They cited inequality in access to financial resources, information, and education and inequality against refugees, women, and children as the fundamental causes. The details of this model will be explained in the next parts of this article.

The characteristics and codes of the interviewees are mentioned in Table 1 and some of their quotes are mentioned in the texts with their codes. However, more examples of their citations are provided in Supplementary Table 2. The researchers’ characteristics are shown in Table 2.

**Table 2:** Characteristic of the researchers.

	FT	PA	AD	HP	AT
Gender	Female	Female	Male	Male	Male
Occupation at the time of study	Researcher	Associate Professor, Dean of the department	Full professor	Associate Professor	Full Professor, Dean of the department
Credentials	Ph.D. candidate	PhD	PhD	PhD	MD MPH PhD
Experience and training	Food policy	Health promotion	Community Nutrition	Community Nutrition	Health policy
Establishment of the relationship before the study	Yes	Yes	Yes	Yes	Yes
Role in Study	Designing the study, Interviewing, conducting data analysis, drafting the article	Data analysis, editing the themes, and finalizing the article	Designing the study, editing themes, and final article	Supervising the study and editing the article	Designing the study, supervising the study, conducting the extraction of themes and model

**A. The mutual relationship between obesity and inequality in Iran**

Interviewed experts believed that obesity seriously threatens disadvantaged groups. They believe that there is a misconception in our society that obesity is a disease of the rich, and this belief can prevent strong support for the implementation of obesity control policies. Interviewees believed that most policymakers think that programs to control other nutritional problems can more effectively meet the needs of lower social groups. “*In my opinion, obesity is not a priority for officials, policymakers or health workers and families. Still underweight is considered by many to be a major problem in our society*.” (AC).

It is strongly suggested that there is a reciprocal relationship between obesity and inequality. It has been repeatedly emphasized that obesity is more common in poor people who have to choose cheap and filling foods. On the other hand, obese children are not accepted in group activities and have less chance for physical activity and education. Obese adults are also less likely to achieve high-paying jobs. This reduces the financial ability of obese people and keeps them in a vicious cycle of poverty and obesity. “*Obese children and adults are caught in a vicious cycle in which obesity breeds poverty and poverty breeds obesity.*. *.. Social acceptance of obese people is influenced by their appearance. Obese people may have less chance of employment because society thinks fat people are untidy and lazy...”* (AM).

**B. The immediate causes of obesity inequality in Iran**

**B.1. Inequality in access to healthy foods**

Participants consistently stated that there are many challenges related to access to healthy foods in low-income families, especially in border and suburban areas. Limited access to nutrient-rich foods, including animal protein sources such as meat, fish, dairy, fruits, and vegetables; has been repeatedly cited as a contributing factor to obesity, particularly in families with less access to resources. Many families are unable to afford healthier foods and are struggling with cellular hunger exacerbated by sanctions and the COVID-19 pandemic. One of the participants said, “*The price of all food, especially healthy foods, is increasing every day due to the sanctions. The Covid-19 pandemic has aggravated this situation. “I am from the middle class and every time I buy less fruit and vegetables. This problem is more serious in families of lower social classes”* (INO1).”

On the other hand, the lower price and accessibility of high-calorie and low-nutrient foods is the reason often cited for the prevalence of obesity and overweight in low-income groups. Subsidizing high-calorie foods and providing inadequate food baskets to poor families exacerbates this situation, as detailed in Part D of this article. *“Obesity is more common in low-income groups than in wealthy people because high-calorie foods are cheaper and more readily available. We subsidize them to some extent. Therefore, they are more economical”* (SSCHFS). “*Low-priced fast foods and snacks are available in every supermarket, even in deprived and rural areas*” (AE).

**B.2. Inequality in access to physical activity facilities**

Less physical and economic access to physical activity equipment in the lower social classes has been repeatedly mentioned as one of the causes of obesity in this group. Some participants cited insufficient social safety as a serious barrier to physical activity, which is more prevalent in peri-urban areas. “*In the suburbs, there are many open spaces and parks, in the prosperous areas of the cities; the density of towers does not leave much open space. Of course, there are many sports clubs in prosperous areas because the high economic power increases the demand for sports*.” (Edu4). “*Suburban parents are more cautious about their child’s physical activity outside the home due to exposure to drugs and other crimes*” (AN2).

Some participants believed that most families cannot afford the high costs of sports clubs. However, few indicated that insufficient physical activity was more likely to be caused by inappropriate beliefs. “*Parents spend a lot of money and time on school theory courses, especially entrance examinations because they believe that academic education plays an important role in the future of their children. We need to make families aware of the importance of physical activity and promote low-cost physical activity such as walking”* (AC1).

Some participants stated that air pollution is another obstacle to physical activity in many Iranian cities. This problem is more serious downtown. In addition, people living in the south of Iran are exposed to dusty weather that limits the possibility of physical activity on most days.

**B.3. Inequality in access to health care services**

Officials in the Ministry of Health insisted that access to health services, especially Primary Health Care (PHC) services, is equal in all parts of the country. On the other hand, most academics believed that there were serious inequalities in access. “*The private health care sector seeks the profits found in affluent cities. Therefore, private health care is concentrated in affluent areas, and the unfortunate reality is that the private sector usually provides high-quality care* (AS1).” Most participants acknowledged that access to obesity treatment facilities, including nutritional counseling and bariatric surgery, is limited to a small number of cities in Iran. In addition, weight management in Iran is expensive and not covered by insurance.

**C. The underlying causes of obesity inequality in Iran**

**C.1. Inequality against children, and women**

Interviewed experts believed that children and women face several limitations that can put them at a higher risk of obesity. Also, our quick review shows that the highest rates of obesity are reported in women from lower socio-economic classes (13, 14). Women are at higher risk of obesity partly due to their physiological issues. By the way, participants entirely indicated that women have substantially lower access to physical activity facilities. They asserted that a limited number of sports clubs are dedicated to ladies, and access is highly restricted in small cities and deprived regions. “*There is less access for women. Even when there are health clubs, the hours dedicated to women are fewer and often inappropriate for working women*. …*Women are not allowed to do certain physical activities in public places. Even if some physical activities are not prohibited by law, people do not accept women who exercise in public places and do not behave properly* (SSCHFS).”

A few participants mentioned the inequality of access to food for women and children. They believed that in the families of the lower social classes and some border towns, the best part of the food was reserved for men. However this issue was not accepted by the majority of interviewees.

Also, some believe that women are not included in the policy-making process. *“Most policies are proposed and formulated by men and they do not have proper information about issues related to children and women. Since obesity disparities are mainly related to women and children. This political approach aggravates these inequalities”* (AS2).

Inefficient financial support for low-income families was a problem identified in this study. “*We often give financial aid and even food baskets to men from poor families and do not monitor the allocation of resources within the household. It is not clear whether this money is allocated to cigarettes,* etc.*, or food and other demands of children... In addition, families are not trained in the proper allocation of financial resources and the selection of appropriate food baskets.” (*AS2*)*.

**C.2. Inequality against refugees**

Our quick review shows that the weight pattern and general health status of refugees in the country are similar to their Iranian neighbors. Unfortunately, most refugees live in disadvantaged areas and face health problems in these areas (18–20). Legal immigrants have access to free PHC and can benefit from all implemented health programs as legal citizens. They can benefit from specialized medical services in public or private clinics or hospitals by paying a fee.” *Legal immigrants are considered full citizens and can benefit from all health services and health-related programs. Unfortunately, many illegal refugees living in Iran face many health problems*…. *We have explained the situation in several meetings with* var*ious government bodies such as the police force, the Ministry of Health, Education and... but it is a complicated issue, many refugees enter our country illegally and we cannot support everyone”* (SSCHFS).

The main problem is social acceptance and negative attitudes against them. Some experts noted that they are not properly accepted and that there are racist reactions against immigrants, mainly from low-income countries. This can reduce their educational and employment opportunities and significantly affect their income. All this endangers their nutritional health.

In addition, refugees face some cultural problems in nutrition. “*Refugees face a cultural gap. Their eating habits are different and they need time to adapt to the new environment, which puts them at risk of malnutrition*” (AM).

**C.3. Inequality in access to information and education**

The experts pointed out that, unfortunately, in schools and media such as the Islamic Republic of Iran Radio and Television, which covers all members of the society, sufficient education about healthy lifestyles is not provided. Therefore, only the upper social classes, who are mostly highly educated, receive detailed information and training.” *A healthy lifestyle should be taught through schools and media that cover the entire society, especially the poor. Schools are the best place to invest in community health*” (AF).

Higher social classes have better education opportunities and also have more access to a variety of information. This creates better job opportunities and more income for them. Higher incomes and better health-related knowledge increase their chances of adopting a healthier diet and lifestyle. This fact can significantly increase the disparity in the prevalence of obesity. “*Inadequate eating habits and other health-related behaviors in children from poor families are not only due to less economic access but also because their parents are less able to teach them about healthy living….. In addition, these children have less access to quality education, which reduces their chances of obtaining good jobs*” (AN1).

**C.4. Inequality in financial resources**

There are not enough opportunities to earn money for families in these underdeveloped areas. This leads to less ability to have a high-quality diet, which leads to an increased risk of obesity, especially in children.” *Job and educational opportunities are much less in the border and deprived areas of the country. Therefore, people are in* var*ious economic and cultural bottlenecks that do not allow them to live a healthy life*” (AM).

In addition, there is a huge inequality in physical access to healthy foods, sports facilities, and health services, which is the result of unbalanced development in different regions of the country. “*They talk over and over about justice and equality, especially when they need our vote. However, there is no equality. Even in the north and south of our province, there is a huge disparity in access to healthy food, sports equipment, and education*” (Edu3).

**D. Do Iranian policies against obesity reduce inequalities?**

Several policies and programs have been approved to improve the health status in Iran, which have a direct or indirect effect on obesity control. These policies are mentioned elsewhere and their achievements and defects are not discussed here. In this article, only the effects of these activities on equality issues are considered, and a list of policy solutions to reduce this inequality is presented in Table 3.

**Table 3:** A policy solution for the causes of obesity inequality in Iran.

Inequality challenge	Policy options to alleviate
Inequality in access to healthy foods	Imposing a tax on unhealthy foods
	Subsidizing healthy foods
	Improving food baskets for needy families
	Allocation of conditional cash transfers to needy families
Inequality in access to physical activity facilities	Improving security in the countryside
	Creating free physical activity facilities in deprived areas
Inequality in access to healthcare	Providing obesity prevention care in PHC.
	Increasing the number of weight management professionals in small towns and rural areas
	Weight management care insurance coverage
Inequality against women, and children	Social marketing for women’s physical activity
	Participation of women and children in policy-making processes
	Increasing facilities and safe places for women’s physical activity
	Monitoring the allocation of cash assistance to families
Inequality against refugees	Increasing the chances of education and especially health-related training for refugees
	Increasing the access of refugees, especially illegal refugees, to healthcare
Inequality in access to information and education	Increasing access to effective health education in schools and mass media
	Increasing access to job skills training, especially in the lower classes
Inequality in access to financial resources	Increasing job opportunities in deprived areas

The most common policy flaw was an excessive focus on treatment rather than prevention. Most of the interviewees believed that prevention has been neglected in Iran’s health system and that excessive attention to treatment favors the rich rather than the poor. “*Our health system is mainly focused on building hospitals and providing medicine. Which vulnerable groups can hospitals help? Hospitals benefit the rich who have more economic power. This problem is exacerbated by the rapid growth of private hospitals”* (AS1).

Iran’s efforts to prevent obesity are mainly focused on educating people about a healthy lifestyle le. Most of the participants, mostly university professors, made it clear that these policies have not only reduced obesity disparities among Iranians but could exacerbate these gaps. Several researchers, particularly participants specializing in the social sciences and economics, have discussed the limited impact of food labeling and nutrition education on the food purchasing habits of low-income households and emphasized the importance of financial issues in family food choices. “*When we educate people, rich people may drink milk instead of soft drinks, for example, but low-income people cannot afford dairy. Dairy price is constantly increasing, and the price is the most important determinant of consumption* (MoHME1)*.”* “*Price matters. Families buy more affordable food, even if it has more total or trans-fats. Perhaps their knowledge or attitude has changed in recent years due to the high volume of nutrition education, but their practice has not*” (Edu4).

An economist noted that we had not established appropriate financial policies. She believed that targeted subside and earmarked taxation could lead the families’ food choices in a healthier direction. She insisted, “*Most foods show a high price elasticity of demand because they are proper substitutes for them. For example, people will easily substitute dough or even water for soft drinks if the price of these unhealthy fattening drinks increases* (AEC).” She and most of the other participants believed that there were no successful policies in this regard. Furthermore, they thought that these policies would most help the lower class of society and reduce health inequalities.. “*Taxation on high-sugar and high-fat foods is recommended by the WHO and other international health organizations... But our governments continue to subsidize sugar and fat in a country where obesity and diabetes are on the rise…Some efforts have been made to tax sugary foods or reformulate foods to reduce sugar and fat. However, they were often unsuccessful due to the non-cooperation of other associations and some deficiencies in upstream legislation.*” (MoHME1).

Reducing or even eliminating subsidies for some healthy foods is another bad policy that our interviewees mentioned several times. They believed that the removal of subsidies for dairy products has led to a significant decrease in dairy consumption in these years. Furthermore, stakeholders repeatedly pointed out that our policies support food production without considering its health effects. “*For more than 28 years, we have been negotiating to subsidize dairy products. We have declared that these dairy products should be subsidized to increase the purchasing power of low-income families. This is an investment in our community that leads to a higher intake of protein and other nutrients. Unfortunately, subsidies have been limited since 2011, we held several negotiations with the “Organization for Targeting Subsidies” and a written notice signed by the Minister of Health was also sent to them. We were not noticed*” (MoHME1).

## Discussion

In the rapid review part of this study, inequality in the prevalence of obesity among Iranians was identified in terms of place of residence, gender, education, and other socio-economic characteristics. Participants in the qualitative part of this study believed that existing inequalities in obesity can significantly affect health disparities and even exacerbate other social and economic inequalities. They added that obesity and inequality are linked through immediate and intermediate causes. Inequality in access to healthy foods, physical activity facilities, and health care are the immediate causes of this inequality. Of course, these immediate factors are caused by intermediate factors, including inequality against women, children, and refugees, and inequality in access to information, education, and financial resources. Legal immigrants enjoy all the facilities of ordinary citizens, but illegal immigrants need special attention.

Energy imbalance is considered the main cause of overweight and obesity, which is explained by high caloric intake and low physical activity (21). In this view, achieving a healthy weight is largely an individual responsibility. Another growing view of obesity is that the obesity epidemic reflects a natural response to an abnormal environment. Cheap, high-calorie foods in large portions are heavily promoted everywhere, creating artificial food environments that lead to high energy consumption. In addition, car-dependent transportation, sedentary work environments, and greater access to passive entertainment combined with less access to physical activity facilities lead to lower energy consumption (9). In other words, our society is full of obesity-promoting factors that overwhelm individual efforts to achieve a healthy weight (9). This imbalance is linked to the lack of supportive policies that define our environment, including the health system, agriculture, transportation, food processing, marketing, education, etc (21). When you have access to healthy choices, personal responsibility can lead to a healthier lifestyle (21). Therefore, it is imperative to support people, especially those of lower socioeconomic status, through sustainable policies that make healthier options more accessible, easier, and more affordable.

The literature review showed the existence of obesity disparities between different geographical areas and sexes. This discrepancy puts people of low socioeconomic groups at an accelerated risk of ischemic heart diseases, cancers, type 2 diabetes, and joint problems, the leading cause of loss of healthy years and premature mortality in lower socioeconomic groups (22). In our rapid review, differences in the prevalence of obesity in Iranian citizens were observed. The literature shows a higher prevalence of obesity in Iranian adults with a low socio-economic base, especially in women, which was also mentioned by the participants in the qualitative part of the study. However the prevalence of childhood obesity in Iran shows a different pattern. Being overweight is more common in more affluent areas, while underweight and short stature are major problems in less developed areas (23, 24). The prevalence of childhood overweight and obesity was higher in prosperous areas of deprived cities in Iran. The prevalence in boys in this city was higher than in girls (25). It should be noted that few participants point to the higher prevalence of underweight in children of low social classes, especially low-income families, which leads to obesity in adulthood. People in low- and middle-income communities are more vulnerable to malnutrition in the early stages of life. Low-income households tend to consume a low-cost diet, which is usually a low-quality diet, high in calories, fat, sugar, and salt, and low in micronutrient density (21). The association between food insecurity with living areas, parents’ education, and income in Iranian society has been established in a recent systematic review (26).

A higher prevalence of obesity in people with low socio-economic levels is a common finding mainly in high-income countries (9). Primarily, they are believed to be more exposed to obesity-promoting factors and less able to prevent weight gain. This fact makes obesity an equity issue (9). Our participants consistently stated that people from lower social classes have limited economic access to healthier, low-calorie, and nutritious foods. Some believe that physical access to healthy foods, especially seasonal access to dairy products, vegetables, and fruits, is severely limited in some deprived areas of Iran. Less access to physical activity facilities was another recurring concept among our participants. They believed that there was limited access to gyms and safe parks in the suburbs.

Inequality against women and children was another recurrently mentioned sketch. Some experts noted that women and children do not participate in policymaking. There were few notions about lower access to nutrient-dense foods for ladies and girls, particularly in privileged areas. However, the most frequent idea was Iranian women’s physical activity limitations. They persistently uttered an urgent need to increase gyms, parks, pools, and other sports venues for girls and ladies. Moreover, some experts propounded those movies or celebrities should publicize various acceptable female physical activities.

In most societies, the prevalence of obesity and overweight is higher in women than in men (27, 28). Physiological situations of women, especially their reproductive roles, seem to be a common reason for this difference (29, 30). However, the magnitude is more robust in countries where injustice against women is more prevalent (31). For example, the prevalence of obesity differs between men and women in the United States by 4%, but in Kuwait by 26% and in South Africa by 29% (31). Several studies showed that the physical activity of girls and not boys decreases sharply in adolescence and remains low in adulthood (32–35). Norms and rules of appropriate behavior impose different restrictions on women’s physical activity in many regions of the world (36, 37). The country’s gender inequality plays a more important role than *per capita* income in creating obesity inequalities. For example, the high level of economic development and wealth of the Persian Gulf countries did not compensate for gender-related health inequalities, including the prevalence of obesity (31). Based on what has been mentioned, policies to reduce obesity may benefit from taking this conjecture into account and a deeper understanding of local gender norms and the institutions that result from them (31).

Another common belief was that refugees had less access to healthier food and health care in Iran. The food insecurity of refugees in Iran has been documented in several studies. It is reported that about 80% of them face some aspect of food insecurity that puts them at risk of obesity (18, 19, 38–40). In illegal refugees, food insecurity is exacerbated (38). The pattern of obesity in Iranian refugees is very similar to families with low social class in Iran, and it is similar to Iranians in their living area (38). Therefore, measures to support the deprived classes of Iran will often solve the problems of legal refugees. But illegal refugees need special attention.

Most of the participants believed that Iran’s efforts to fight obesity are mainly focused on educational issues, which benefit the most educated and upper social classes. They repeatedly announced that despite the strong efforts of the Ministry of Health, more effective solutions such as taxes on high-calorie, high-fat, and sweet foods are not implemented in Iran. It also pointed out the need to implement policies to provide healthier foods such as vegetables, fruits, and milk in schools and low-income families.

Studies conducted in other countries have shown similar approaches to obesity control, and usually, insufficient attention has been paid to reducing inequality in obesity control. Recently, several international and national health organizations have announced the urgent need to redesign obesity control programs with a more equity-oriented approach. They reported that even in well-designed weight loss interventions, people were unable to maintain a healthy weight for a long. Being in fattening environments, especially in the lower socioeconomic classes, makes it extremely difficult for a person to maintain a proper weight. Providing affordable healthy foods and creating an environment that supports physical activity, especially in disadvantaged areas; have been cited as key strategies to effectively control obesity (41–43).

According to the above, it can be concluded that the slope of inequality in obesity is toward low socio-economic groups. Many direct and indirect causes are creating this inequality, and incorrect obesity control policies exacerbate it. There is an urgent need to redesign these health policies as well as other policies of the country to reduce these inequalities. Stakeholders in obesity control emphasized that we should modify our health policies in line with justice. They mentioned that we should urgently modify some policies to ameliorate the inequality in access to healthy foods, health care, and physical activity facilities. Better access to healthy foods could be reached by more emphasis on taxing unhealthy foods, subsidizing healthy foods, providing free healthy meals in schools, especially in disadvantaged areas, and providing nutrient-dense foods to low-income families. These policies can not only reduce the risk of obesity but also reduce inequalities.

Environmental re-engineering to increase opportunities for physical activity, especially in marginalized and disadvantaged areas of the country, was one of the justice-oriented policies to control obesity in Iran. We need to pay particular attention to providing higher access to physical activity facilities for girls and ladies. Better and more serious physical activity programs in schools and universities could enhance their access to some extent. However, more fundamental actions such as providing athlete female models and removing some legal and cultural boundaries are needed.

Inequality in access to health care was another immediate cause of obesity in equity. According to stakeholders, we need to provide better preventive health care, especially in underserved areas. In addition, preventive health care, including nutritional and psychological counseling, should be covered by insurance to ensure access to low-income families.

The mentioned policies will reduce the obesity inequalities in Iran, but for a long-lasting improvement, there is a need to implement more fundamental policies that lead to the reduction of inequality in access to educational facilities and job opportunities. Also, more participation for women should be provided in policy-making and children’s voices and needs should be heard.

### Rigor of study

This study is the first study that explains the cause of obesity inequality in Iran. It provides several practical policy recommendations that can help improve this health-related inequality in Iran and other countries. We hope this recommendation is practical as a wide range of stakeholders are included in our study. However, the results of this study should be further evaluated through some experimental interventions. In particular, the effectiveness and cost-effectiveness of this policy option should be investigated.

This study had several limitations related to the qualitative method. However, we applied the Lincoln and Guba criteria to ensure validity (44). In this way, a detailed description of the study process was provided at each stage. Also, the validity and consistency of the data were verified in three main ways, long and in-depth interaction with the participants, review by the participants, and review by faculty members. The main strength of our study is the interview with all stakeholders, including academics, industries, governments, and civil societies; which provided maximum diversity of the sample. This approach can confirm the relevance and validity of the findings and the possibility of clarifying the research question in different aspects.

The presence of a consistent interviewer in all interviews and spending enough time to collect accurate data can ensure reliability to some extent. Five faculty members reviewed the methods and findings of the study to establish reliability and trustworthiness. Modification of the interview guide by expert faculty members increased the validity of our findings.

### Policy implications

The high prevalence of obesity in Iran has turned it into a serious health problem that hinders Iran’s efforts to achieve a higher HDI. Obesity control policies should pay more attention to solving existing inequalities in obesity. Increasing access to healthy foods at low prices and free physical activity facilities in disadvantaged areas, taxes on unhealthy foods, and subsidizing healthy foods were suggested as ways to reduce these inequalities. In addition, providing healthy meals to schools, especially in disadvantaged areas, and providing nutrient-dense meals to low-income families can increase the quality of food for vulnerable people. Expanding safe walking or cycling routes, especially in the suburbs, and creating more free gyms for women are effective policies to increase physical activity that should be implemented. In addition, policymakers should improve access to health education and early weight control counseling across the country.

## Conclusion

Obesity control is a central part of NCD control and no country can achieve the SDH goals without proper obesity management. Stakeholders with different expertise participating in this study emphasized that to manage this fundamental health issue, major strategies that target the underlying causes of obesity, including disparities, must be considered. There is a need to integrate efforts to build social capacities and reduce the factors that prevent a healthy lifestyle, especially in socially disadvantaged groups.

## Data availability statement

The raw data supporting the conclusions of this article will be made available by the authors, without undue reservation.

## Ethics statement

The Ethics Committee of Tehran University of Medical Sciences approved the protocol of this project (No. 97-03-161-40986). All procedures in all phases of our study were performed following the ethical guidelines of the Tehran University of Medical Sciences. In addition, we followed the Chatham House Rule and did not report any data with participants’ names. After explaining the aims and protocol of the study, informed consent was obtained from all participants. Permission was obtained to record the interview. They were assured that none of their comments would be published under their names and that no one but the original research team would have access to the interviews. They can leave the study at any time.

## Author contributions

AT, FT, and AD designed the study. FT conducted the interviews and their initial analysis. HP supervised the sampling and interview process. PA reviewed the analysis and coding process. Model construction and manuscript drafting were performed by FT under the close supervision of AT. All authors contributed to the article and approved the submitted version.
