# Supplementary material for: Worse becomes the worst: obesity inequality, its determinants and policy options in Iran
Source: Front Public Health. 2024 Feb 7;12:1225260. doi: 10.3389/fpubh.2024.1225260 (PMC10880032; doi:10.3389/fpubh.2024.1225260)
Supplement: Supplementary file 2 [file Data_Sheet_1.docx]

| **Supplementary Table1. A rapid review of obesity inequality in Iran** | | | | |
| --- | --- | --- | --- | --- |
| author | year | reference | Methods | Results |
| Khazaei S | 2017 | [[45](#_ENREF_45)] | A systematic review of 51 articles on the prevalence of obesity in Iranian children aged 6 to 18 years. | The prevalence of obesity was 82.5% (CI: 5.6-5.66: 95%) in Iranian students. The prevalence of obesity was higher in boys (85.6% versus 13.5%). The prevalence was different in the provinces and the highest was reported in the northern and northwest regions. |
| Bakhshi E | 2015 | [[46](#_ENREF_46)] | Data analysis of the STEP survey in 2011, which was conducted on 8639 adults in Iran under the supervision of the World Health Organization. | The prevalence of obesity was 3.22%. After adjusting for covariates, obesity was associated with being female (OR 1.47; 95% CI: 1.26–1.72), urban residence (OR 1.54; 95% CI, 1.1–1.80), and unemployment and low education (OR 0.68, respectively). The highest prevalence of obesity was in the Turk-Gilk-Ler-Turkmen ethnic group (25.5 percent), followed by Fars (21.5 percent) and others (16.9 percent). |
| Khashayar P | 2018 | [[25](#_ENREF_25)] | Data analysis of “The fourth phase of childhood and Adolescence Surveillance and Prevention of Adult Non-communicable disease” on 14,880 students in 2010-2019 in Iran based on the GSHS method. | The prevalence of overweight and obesity was reported as 7.9 and 9.11. In the multivariable-adjusted model, obesity was associated with being a boy (OR: 1.58), having high SES (OR: 1.89), and living in urban areas (OR: 1.58). |
| Kelishadi | 2014 | [[47](#_ENREF_47)] | Meta-analysis of 107 studies on the prevalence of obesity in Iranian children. | The prevalence of overweight and obesity in childhood is about 1.5% (95% CI, 8.4-4.5) and 10.8% (95% CI, 2.10-4.11) and remained relatively stable during the 2000s. Their prevalence did not show significant differences in terms of gender and age. |
| Bahreynian M | 2014 | [[23](#_ENREF_23)] | Data analysis of the CASPIAN-II surveys, a national cross-sectional survey conducted in 2011-2012. | Among the 5,624 adolescents aged 10–18 years who participated in this study, obesity was more common in higher SES and underweight and short stature were more common in lower SES. The average BMI was significantly lower in the southeast and the highest BMI was observed in the center of the country. However, there was no significant difference in waist circumference. |
| Amin R | 2021 | [[48](#_ENREF_48)] | Data from 28,321 adults participating in the STEP 2016 study were used. | The prevalence of obesity and pre-obesity was 60.3%. The prevalence of pre-obesity was higher in men (39%) than in women, but the prevalence of obesity was twice as high in women (4.30% vs. 6.15%). The overall prevalence of overweight (obesity/pre-obesity) was very different in different provinces and varied between 40.8 and 70.1 percent. The highest prevalence of pre-obesity was reported in Alborz (8.40%) and Ardabil (7.40%), and the highest prevalence of obesity was reported in Semnan (6.30%) and West Azerbaijan (0.3%). The prevalence of obesity was negatively related to higher education levels, being male, and employment. |
| Djalalnia S | 2015 | [[13](#_ENREF_13)] | Meta-analysis of 119 studies attributed to 146,596 individuals on the prevalence of obesity in Iranian adults. | Older age (30< VS >30, OR 1.66; 95% CI 1.22.22), higher education (more than 12 years; OR 0.6, 95% CI: 0.43-0 /83), being married (OR 2/14; 95% CI 1/36-3/38), residence in urban areas (OR 1/58; 95% CI 1/18-2/11) and female gender (OR 2/06; 95% CI 1/45-2/93) were associated with obesity. |
| amin R | 2021 | [[49](#_ENREF_49)] | A cross-sectional study on the anthropometric indices of 2-5-year-old urban children from eight different cities of Iran. Nutritional status was assessed with WHO anthro software and family food security was assessed by HFIAS. | Food security was significantly associated with higher BMI and shorter height. |
| Emamian MH | 2017 | [[14](#_ENREF_14)] | The data of 89,400 people aged 15 to 64 participating in the “Iran Surveillance System for the risk factor of non-communicable diseases in 2005” was analyzed. | The inequality slope index for obesity was -13.1 (95% CI -16.3 to -9.8) percentage units and the concentration index was -0.123. The level of inequality varied greatly between provinces and was more severe in women and urban residents. The prevalence of obesity was higher in low SES (2.20%; 95% CI: 9.4-20.19) than in high SES groups (0.11%; 95% CI: 6.11-5.10). More than 90% of this gap was due to age, gender, and marital status. |

| **Supplementary Table2. Examples of interviewees’ quotes** | | | |
| --- | --- | --- | --- |
| Interviewee's code | Quote | Sub-theme | **Theme** |
| AC | *In my opinion, obesity is not a priority for officials, policymakers health workers, and families. Still, being underweight is considered by many to be a major problem in our society.* | Not recognizing obesity as an important health issue | **The Mutual Relationship between Obesity and Inequality in Iran** |
| AM | *The problem is that there is no strong political will at the local and national levels to control obesity.* | Lack of political will |  |
| MOHME1 | *There is a prompt need to advocate for changing the attitude of high-ranking officials. They don't consider obesity as a serious health matter. They presume it is a problem of prosperity in high social class groups.* | Obesity is a problem of prosperity |  |
| AM | *Obese children and adults are caught in a vicious cycle in which obesity breeds poverty and poverty breeds obesity… Social acceptance of obese people is influenced by their appearance. Obese people may have less chance of employment because society thinks fat people are untidy and lazy* | *The vicious cycle* of obesity and inequality |  |
| Edu3 | *They talk over and over about justice and equality, especially when they need our votes. However, there is no equality. Even in the north and south of our province, there is a huge disparity in access to healthy food, sports equipment, and education* | Inequality in access to healthy food | **Inequality in access to healthy foods** |
| INO1 | *The price of all food, especially healthy foods, is increasing every day due to the sanctions. The Covid-19 pandemic has aggravated this situation. I am from the middle class and every time I buy less fruit and vegetables. This problem is more serious in families of lower social classes* | The higher prices of healthy food |  |
| SSCHFS | *Obesity is more common in low-income groups than in wealthy people because high-calorie foods are cheaper and more readily available. We subsidize them to some extent. Therefore, they are more economical* | high affordability of unhealthy foods |  |
| AE | *Low-priced fast foods and snacks are available in every supermarket, even in deprived and rural areas* | More fast-food restaurants in slum areas |  |
| Edu4 | *In the suburbs, there are many open spaces and parks, and in the prosperous areas of the cities; the density of towers does not leave much open space. Of course, there are many sports clubs in prosperous areas because the high economic power increases the demand for sports*. | Unequal distribution of parks, gyms, clubs, and bike, and walking paths | **Inequality in access to physical activity facilities** |
|  |  | The high density of buildings in prosperous areas of the city |  |
| AC1 | *Parents spend a lot of money and time on school theory courses, especially entrance examinations because they believe that academic education plays an important role in the future of their children. We need to make families aware of the importance of physical activity and promote low-cost physical activity such as walking.* | Low importance of physical activity |  |
| AN2 | *Suburban parents are more cautious about their child's physical activity outside the home due to exposure to drugs and other crimes* | Concerns about the safety of children |  |
| MOHME2 | *Health care services, especially PHC services, which are free for all, are provided in rural and deprived provinces even better than in the capital.* | High access to PHC | **Inequality in access to health care services** |
| MOHME3 | *PHC is provided for all. We even have mobile teams to take care of the nomads, especially for screening and vaccination.* |  |  |
| AS1 | *The private healthcare sector seeks the profits found in affluent cities. Therefore, private health care is concentrated in affluent areas, and the unfortunate reality is that the private sector usually provides high-quality care* | Low access to high-quality care |  |
| Edu3 | Even in some big cities, there are only a few nutrition counseling offices. Nutrition counseling is considered luxurious and not necessarily cared for in our society. | No access to dietitians in deprived areas |  |
| INT1 | There are not enough obesity clinics across the country. In particular, government clinics do not cover obesity-related surgeries, except for a few hospitals in Tehran, the capital of Iran. | Lack of obesity treatment in deprived areas |  |
| MOHME2 | Access to these facilities is lower for ladies. We don’t have proper places for ladies’ and girls' physical activity in the capital city. We don't have any in small cities. | Low chance of physical activity for women | **Inequality against women and children** |
| SSCHFS | *There is less access for women. Even when there are health clubs, the hours dedicated to women are fewer and often inappropriate for working women*. …*Women are not allowed to do certain physical activities in public places. Even if some physical activities are not prohibited by law, people do not accept women who exercise in public places and do not behave properly* | The cultural barrier to women's physical activity |  |
| AS2 | *Most policies are proposed and formulated by men and they do not have proper information about issues related to children and women. Since obesity disparities are mainly related to women and children. This political approach aggravates these inequalities*  *What is happening is the result of linear, wrong, and ideological policy-making processes, which are mainly carried out by men without a life-cycle approach and regard for the wishes of women and children.* | Not participating women and children in the policy-making process |  |
| AS2 | *We often give financial aid and even food baskets to men from poor families and do not monitor the allocation of resources within the household. It is not clear whether this money is allocated to cigarettes, etc., or food and other demands of children... In addition, families are not trained in the proper allocation of financial resources and the selection of appropriate food baskets.* | Inappropriate cash transferring |  |
| AEC | The allocation of donated food baskets to family members is not properly monitored. In some parts of Iran, especially in low social class families, sons are considered superior. These meals can be allocated to men and boys rather than malnourished children. | Inappropriate cash transferring |  |
| AM | *Refugees face a cultural gap. Their eating habits are different and they need time to adapt to the new environment, which puts them at risk of malnutrition* | Cultural lag of refugees | **Inequality against refugees** |
| AN2 | There are racist reactions against refugees even in our universities and we have not done any effective action to reduce it. | Racism against refugees |  |
| SSCHFS | *Legal immigrants are considered full citizens and can benefit from all health services and health-related programs. Unfortunately, many illegal refugees living in Iran face many health problems We have explained the situation in several meetings with various government bodies such as the police force, the Ministry of Health, Education, and... but it is a complicated issue, many asylum seekers enter our country illegally and we cannot Let's support everyone* | low access to health care for illegal refugees |  |
| AN2 | Legal immigrants have access to free PHC but it seems that they cannot afford specialized services and even healthy foods just like other lower-income families in our society. | The unaffordability of specialized care |  |
| AEC | Information is a public good that should be provided by governments. Unfortunately, there is not equal access to information about healthy foods and physical activity. Some people don't even know where and how they can exercise freely and for free. | Unequal access to information | **Inequality in access to education and information** |
| AM | Nutritional labels on food products can lead to better food choices if the consumer is properly educated. Reading and understanding these labels is only possible for highly educated people. | Improper health education |  |
| AN1 | *Inadequate eating habits and other health-related behaviors in children from poor families are not only due to less economic access but also because their parents are less able to teach them about healthy living. In addition, these children have less access to quality education, which reduces their chances of obtaining good jobs* | Unequal access to education |  |
| AF | *A healthy lifestyle should be taught through schools and media that cover the entire society, especially the poor. Schools are the best place to invest in community health* | Ineffective health education |  |
| AM | *Job and educational opportunities are much less in the border and deprived areas of the country. Therefore, people are in various economic and cultural bottlenecks that do not allow them to live a healthy life* | Low job opportunities in deprived areas | **Inequality in access to financial resources** |
|  |  | Income inequality |  |
| Edu3 | *They talk over and over about justice and equality, especially when they need our votes. However, there is no equality. Even in the north and south of our province, there is a huge disparity in access to healthy food, sports equipment, and education* | Regional inequality in access to resources |  |
| MOHME3 | We pay more attention to treatment rather than prevention. We are eager to screen children and families in school or the PHC system and we neglect educating and training families to prevent obesity. | Low priority of prevention | **Do Iranian policies against obesity reduce inequalities?** |
| AS1 | *Our health system is mainly focused on building hospitals and providing medicine. Which vulnerable groups can hospitals help? Hospitals benefit the rich who have more economic power. This problem is exacerbated by the rapid growth of private hospitals* | Excessive attention to treatment |  |
| edu2 | Several justice-oriented programs related to improving the health of students have been approved, including free milk in schools, healthy buffets, and student screening. Unfortunately, these programs are not properly implemented and evaluated. | Improper implementation |  |
| MoHME1 | *When we educate people, rich people may drink milk instead of soft drinks, for example, but low-income people can't afford dairy. Dairy price is constantly increasing, and the price is the most important determinant of consumption* | Education could result in change in rich families |  |
| Edu4 | *Price matters. Families buy more affordable food, even if it has more total or trans fat. Perhaps their knowledge or attitude has changed in recent years due to the high volume of nutrition education, but their practice has not* |  |  |
| MoHME1 | *Taxation on high-sugar and high-fat foods is recommended by the WHO and other international health organizations... But our governments continue to subsidize sugar and fat in a country where obesity and diabetes are on the rise…Some efforts have been made to tax sugary foods or reformulate foods to reduce sugar and fat. However, they were often unsuccessful due to the non-cooperation of other associations and some deficiencies in upstream legislation.* |  |  |
| AEC | *Most foods show a high price elasticity of demand because they are proper substitutes for them. For example, people will easily substitute dough or even water for soft drinks if the price of these unhealthy fattening drinks increases.* | Improper pricing policy |  |
| FD | We made a strong effort to modify the food standards to make them less unhealthy and obesogenic. Although we were able to reduce the sugar of some drinks and cereals, there is a long way ahead. We need a better collaboration with the Institute of Standards & Industrial Research of Iran to modify food standards. | Improper food Standards |  |
| SSCHFS | We considered rice, sugar, and oils as basic commodities in our upstream rules which condemn putting a tax on them. | No tax on unhealthy foods |  |
| MoHME1 | *We have declared that these dairy products should be subsidized to increase the purchasing power of low-income families. This is an investment in our community that leads to a higher intake of protein and other nutrients...Unfortunately, subsidies have been limited since 1390, we held several negotiations with the "Organization for Targeting Subsidies" and a written notice signed by the Minister of Health was also sent to them. We were not noticed”* | Removing subsidies for some healthy foods |  |

Supplementary Material References :

1. *Obesity and overweight*. [fact sheet] 2021/10/14]; Available from: <https://www.who.int/news-room/fact-sheets/detail/obesity-and-overweight>.

2. World Health Organization. The State of Food Security and Nutrition in the World 2022: Repurposing food and agricultural policies to make healthy diets more affordable. Food & Agriculture Org.; 2022 Jul 6.

3. Popkin, B.M., L.S. Adair, and S.W. Ng, *Global nutrition transition and the pandemic of obesity in developing countries.* Nutrition Reviews, 2012. 70(1): p. 3-21. https://doi.org/10.1111/j.1753-4887.2011.00456.x

4. Musaiger, A.O., and H.M. Al-Hazzaa, *Prevalence and risk factors associated with nutrition-related noncommunicable diseases in the Eastern Mediterranean region.* International journal of general medicine, 2012. 5: p. 199. <https://doi.org/10.2147/IJGM.S29663>.

5. Bakhtiari, A., et al., *Assessment and prioritization of the WHO “best buys” and other recommended interventions for the prevention and control of non-communicable diseases in Iran.* BMC Public Health, 2020. 20(1): p. 1-16. https://doi.org/10.1186/s12889-020-8446-x

6. Robertson, A. and B. Loring, *Obesity and inequities. Guidance for addressing inequities in overweight and obesity*. 2014: World Health Organization. Genova.

7. Hoffmann, R., et al., *Obesity and the potential reduction of social inequalities in mortality: evidence from 21 European populations.* The European Journal of Public Health, 2015. 25(5): p. 849-856. <https://doi.org/10.1093/eurpub/ckv090>

8. Kulhánová, I., et al., *The role of three lifestyle risk factors in reducing educational differences in ischaemic heart disease mortality in Europe.* The European Journal of Public Health, 2017. 27(2): p. 203-210. <https://doi.org/10.1093/eurpub/ckw104>

9. Kumanyika, S., *Getting to equity in obesity prevention: A new framework.* NAM Perspectives, USA. 2017.

10. Swinburn, B.A., et al., *The global syndemic of obesity, undernutrition, and climate change: the Lancet Commission report.* The Lancet, 2019. 393(10173): p. 791-846. https://doi.org/10.1016/S0140-6736(18)32822-8

11. Klerings, I., et al., *Rapid reviews methods series: Guidance on literature search.* BMJ Evidence-Based Medicine, 2023. <https://doi.org/10.1136/bmjebm-2022-112079>

12. Garritty, C., et al., *Cochrane Rapid Reviews Methods Group offers evidence-informed guidance to conduct rapid reviews.* Journal of Clinical Epidemiology, 2021. 130: p. 13-22. <https://doi.org/10.1016/j.jclinepi.2020.10.007>

13. Djalalinia, S., et al., *Inequality of obesity and socioeconomic factors in Iran: a systematic review and meta-analyses.* Medical journal of the Islamic Republic of Iran, 2015. 29: p. 241.

14. Emamian, M.H., et al., *Obesity and its socioeconomic determinants in Iran.* Economics & Human Biology, 2017. 26: p. 144-150. <https://doi.org/10.1016/j.ehb.2017.03.005>.

15. Esmaeily, H., et al., *Association between socioeconomic factors and obesity in Iran.* Pak J Nutr, 2009. 8(1): p. 53-56.

16. Najafi, F., et al., *Measuring and decomposing socioeconomic inequalities in adult obesity in Western Iran.* Journal of Preventive Medicine and Public Health, 2018. 51(6): p. 289. doi: [10.3961/jpmph.18.062](https://doi.org/10.3961%2Fjpmph.18.062)

17. Raeisi, A., et al., *Socioeconomic inequality of overweight and obesity of the elderly in Iran:*

*Bushehr Elderly Health (BEH) Program.* BMC Public Health, 2017. 17(1): p. 72. https://doi.org/10.1186/s12889-016-3912-1

18. Abdollahi, M., et al., *High occurrence of food insecurity among urban Afghan refugees in Pakdasht, Iran 2008: a cross-sectional study.* Ecology of food and nutrition, 2015. 54(3): p. 187-199. <https://doi.org/10.1080/03670244.2013.834819>.

19. Omidvar, N., et al., *Food insecurity and its sociodemographic correlates among Afghan immigrants in Iran.* Journal of health, population, and nutrition, 2013. 31(3): p. 356. doi: [10.3329/jhpn.v31i3.16828](https://doi.org/10.3329%2Fjhpn.v31i3.16828)

20. Pakravan-Charvadeh, M.R., H. Vatanparast, and C. Flora, *Food Insecurity Status of Afghan Refugees is Linked to Socioeconomic and Resettlement Status, Gender Disparities and Children's Health Outcomes in Iran.* Child Indicators Research, 2021: p. 1-22. https://doi.org/10.1007/s12187-021-09827-y.

21. *Obesity and overweight*. [fact sheet] 2021 2021/10/14]; Available from: <https://www.who.int/news-room/fact-sheets/detail/obesity-and-overweight>.

22. Robertson, A., T. Lobstein, and C. Knai, *Obesity and socio-economic groups in Europe: Evidence review and implications for action. 2007.* Brussels: European Commission, report for DG Sanco, 2013.

23. Bahreynian, M., et al., *Prevalence of growth disorders in a nationally representative sample of Iranian adolescents according to socioeconomic status: the CASPIAN-III Study.* Pediatrics & Neonatology, 2015. 56(4): p. 242-247. <https://doi.org/10.1016/j.pedneo.2014.12.001>

24. Moradi, S., et al., *Food insecurity and the risk of undernutrition complications among children and adolescents: A systematic review and meta-analysis.* Nutrition, 2019. 62: p. 52-60. <https://doi.org/10.1016/j.nut.2018.11.029>

25. Khashayar, P., et al., *Childhood overweight and obesity and associated factors in iranian children and adolescents: a multilevel analysis; the CASPIAN-IV study.* Frontiers in pediatrics, 2018. 6: p. 393. <https://doi.org/10.3389/fped.2018.00393>

26. Narmcheshm, S., et al., *Socioeconomic Determinants of Food Insecurity in Iran: A Systematic Review.* Journal of Asian and African Studies, 2023: April.17: 00219096231161893. <https://doi.org/10.1177/00219096231161893>.

27. Finucane, M.M., et al., *National, regional, and global trends in body-mass index since 1980: systematic analysis of health examination surveys and epidemiological studies with 960 country-years and 9· 1 million participants.* The lancet, 2011. 377(9765): p. 557-567.

28. WHO. *Data for saving lives 2010 Available from* [*https://apps*](https://apps/)*. WHO. int/infobase*. Index. aspx 2010.

29. Brooks, R. and A. Maklakov, *Sex differences in obesity associated with total fertility rate.* PLoS One, 2010. 5(5): p. e10587. <https://doi.org/10.1371/journal.pone.0010587>.

30. Newby, P.K., et al., *Early anthropometric measures and reproductive factors as predictors of body mass index and obesity among older women.* International journal of obesity, 2005. 29(9): p. 1084-1092. https://doi.org/10.1038/sj.ijo.0802996

31. Garawi, F., et al., *Global differences between women and men in the prevalence of obesity: is there an association with gender inequality?* European journal of clinical nutrition, 2014. 68(10): p. 1101-1106. https://doi.org/10.1038/ejcn.2014.86

32. Bauman, A., et al., *The international prevalence study on physical activity: results from 20 countries.* International journal of behavioral nutrition and physical activity, 2009. 6(1): p. 1-11. https://doi.org/10.1186/1479-5868-6-21.

33. Owen, C.G., et al., *Ethnic and gender differences in physical activity levels among 9–10-year-old children of white European, South Asian and African–Caribbean origin: the Child Heart Health Study in England (CHASE Study).* International journal of epidemiology, 2009. 38(4): p. 1082-1093. <https://doi.org/10.1093/ije/dyp176>.

34. Ridgers, N.D., et al., *Examining influences on boy’s and girls’ physical activity patterns: the A-CLASS project.* Pediatric exercise science, 2010. 22(4): p. 638-650. DOI:  <https://doi.org/10.1123/pes.22.4.638>

35. Trost, S.G., et al., *Age and gender differences in objectively measured physical activity in youth.* Medicine and science in sports and exercise, 2002. 34(2): p. 350-355.

36. Krieger, N., *Genders, sexes, and health: what are the connections—and why does it matter?* International journal of epidemiology, 2003. 32(4): p. 652-657. <https://doi.org/10.1093/ije/dyg156>

37. Sen, G., P. Östlin, and A. George, *Unequal, Unfair, Ineffective and Inefficient: Gender Inequity in Health: why it Exists and how We Can Change it: Final Report to the WHO Commission on Social Determinants of Health*. 2007: World Health Organization. Karolinska Institute. Stockholm.

38. Kamali, Z., et al., *An Overview of Food Security Statuses in Afghan Refugees in Iran.* Nutrition and Food Sciences Research, 2021. 8(2): p. 5-9. DOI: [10.52547/nfsr.8.2.5](http://dx.doi.org/10.52547/nfsr.8.2.5).

39. Khakpour, M., et al., *The Association Between Afghan Refugees’ Food Insecurity and Socio-economic Factors in Iran: A Case Study of Khorasan Razavi Province.* Border Crossing, 2021. 11(1): p. 51-66. DOI:  <https://doi.org/10.33182/bc.v11i1.1220>

40. Maarefvand, M. and S.Z. Morshedi, *Afghan Immigrant Women's Food Security in Farahzad and Shahrerey.* Quarterly journal of social work, 2016. 5(1): p. 34-40.( full article in persian) URL:http://socialworkmag.ir/article-1-163-en.html

41. Mattes, R. and G.D. Foster, *Food environment and obesity.* Obesity, 2014. 22(12): p. 2459-2461.   <https://doi.org/10.1002/oby.20922>

42. Drewnowski, A., *The economics of food choice behavior: why poverty and obesity are linked*, in *Obesity treatment and prevention: new directions*. 2012, Karger Publishers. Berlin. p. 95-112. DOI: <https://doi.org/10.1159/000341303>

43. Kumanyika, S.K., *A framework for increasing equity impact in obesity prevention.* American Journal of Public Health, 2019. 109(10): p. 1350-1357. [https://doi.org/10.2105/AJPH.2019.305221](https://ajph.aphapublications.org/doi/abs/10.2105/AJPH.2019.305221)

44. Lincoln, Y.S., *Naturalistic inquiry.* The Blackwell encyclopedia of sociology, 2007.
